# Somatic mutations and copy number variations in breast cancers with heterogeneous *HER2* amplification

**DOI:** 10.1002/1878-0261.12650

**Published:** 2020-03-05

**Authors:** Mieke R. Van Bockstal, Marie Colombe Agahozo, Ronald van Marion, Peggy N. Atmodimedjo, Hein F. B. M. Sleddens, Winand N. M. Dinjens, Lindy L. Visser, Esther H. Lips, Jelle Wesseling, Carolien H. M. van Deurzen

**Affiliations:** ^1^ Department of Pathology Erasmus MC Cancer Institute Rotterdam The Netherlands; ^2^ Division of Molecular Pathology The Netherlands Cancer Institute Amsterdam The Netherlands; ^3^ Department of Pathology The Netherlands Cancer Institute Amsterdam The Netherlands

**Keywords:** breast cancer, copy number variations, HER2 amplification, intratumour heterogeneity, next‐generation sequencing, somatic mutation

## Abstract

Intratumour heterogeneity fuels carcinogenesis and allows circumventing specific targeted therapies. *HER2* gene amplification is associated with poor outcome in invasive breast cancer. Heterogeneous *HER2* amplification has been described in 5–41% of breast cancers. Here, we investigated the genetic differences between HER2‐positive and HER2‐negative admixed breast cancer components. We performed an in‐depth analysis to explore the potential heterogeneity in the somatic mutational landscape of each individual tumour component. Formalin‐fixed, paraffin‐embedded breast cancer tissue of ten patients with at least one HER2‐negative and at least one HER2‐positive component was microdissected. Targeted next‐generation sequencing was performed using a customized 53‐gene panel. Somatic mutations and copy number variations were analysed. Overall, the tumours showed a heterogeneous distribution of 12 deletions, 9 insertions, 32 missense variants and 7 nonsense variants in 26 different genes, which are (likely) pathogenic. Three splice site alterations were identified. One patient had an *EGFR* copy number gain restricted to a HER2‐negative *in situ* component, resulting in EGFR protein overexpression. Two patients had *FGFR1* copy number gains in at least one tumour component. Two patients had an 8q24 gain in at least one tumour component, resulting in a copy number increase in *MYC* and *PVT1*. One patient had a *CCND1* copy number gain restricted to a HER2‐negative tumour component. No common alternative drivers were identified in the HER2‐negative tumour components. This series of 10 breast cancers with heterogeneous *HER2* gene amplification illustrates that HER2 positivity is not an unconditional prerequisite for the maintenance of tumour growth. Many other molecular aberrations are likely to act as alternative or collaborative drivers. This study demonstrates that breast carcinogenesis is a dynamically evolving process characterized by a versatile somatic mutational profile, of which some genetic aberrations will be crucial for cancer progression, and others will be mere ‘passenger’ molecular anomalies.

## Graphical abstract

This in‐depth analysis of 10 breast cancers with heterogeneous *HER2* gene amplification illustrates that HER2 positivity is not an unconditional prerequisite for the maintenance of tumour growth. This study demonstrates that breast carcinogenesis is a dynamically evolving process characterized by a versatile somatic mutational profile: some genetic aberrations will be crucial for cancer progression, and others will be mere ‘passenger’ molecular anomalies.

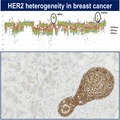

**Abbreviations**
ASCO/CAP: American Society of Clinical Oncology/College of American Pathologists
CNV: copy number variation
DCIS: ductal carcinoma *in situ*
ER: oestrogen receptor
FEDERA: Federation of Dutch Medical Scientific Societies
FFPE: formalin‐fixed, paraffin‐embedded
ILC: invasive lobular carcinoma
LCIS: lobular carcinoma *in situ*
lncRNA: long noncoding RNA
NGS: next‐generation sequencing
NST: no special type
PR: progesterone receptor
SNP: single nucleotide polymorphism

## 1. Introduction

Cancer is a genetic disease, resulting from an accumulation of successive somatic gene mutations that drive cancer cell proliferation (Tomasetti *et al.*, 2017). Invasive breast cancer is heterogeneous and comprises different molecular subtypes (Perou *et al.*, 2000). Around 12–20% of invasive breast cancers have a *HER2* gene amplification, which generally results in overexpression of the HER2 protein (Kraus *et al.*, 1987; Ross, 2010; Venter *et al.*, 1987). The *HER2* gene, located at 17q12‐21, encodes a 185 kDa transmembrane tyrosine kinase receptor. The HER2 receptor has no known ligand of its own but activates other receptors of the HER family by heterodimerization (Barros *et al.*, 2010). *HER2* amplification is associated with shorter disease‐free and overall survival in patients with node‐negative and node‐positive invasive breast cancer treated with adjuvant chemotherapy and/or local radiation (Slamon *et al.*, 1987, 1989). With the advent of the humanized monoclonal anti‐HER2 antibody trastuzumab (Herceptin; Genentech, San Francisco, CA, USA), HER2 has evolved from a mere prognostic marker to a predictive marker and a target for therapy (Ross and Fletcher, 1999). Since then, the anti‐HER2 treatment arsenal has substantially expanded, and current therapeutic options include trastuzumab, pertuzumab (Perjeta; Genentech), trastuzumab emtansine or T‐DM1 (Kadcyla; Genentech) and lapatinib (Tykerb; GlaxoSmithKline, Brentford, UK).

Most HER2‐positive carcinomas, both *in situ* and invasive, present with homogeneous HER2 overexpression and amplification, implying that it is a key molecular event that propels cancer cell proliferation. Such genetic events occur early in the process of carcinogenesis and are designated ‘truncal’ somatic events (McGranahan *et al.*, 2015). However, an intratumoral heterogeneous pattern of *HER2* amplification is not uncommon. Heterogeneity has been described in 5–41% of HER2‐positive breast cancers, depending on its definition (Cottu *et al.*, 2008; Ng *et al.*, 2015; Ohlschlegel *et al.*, 2011). The latest ASCO/CAP guidelines do not define intratumour heterogeneity (Wolff *et al.*, 2018), but previous studies discerned regional from genetic heterogeneity (Bartlett *et al.*, 2011; Hanna *et al.*, 2014; Seol *et al.*, 2012; Vance *et al.*, 2009). Genetic *HER2* heterogeneity is defined as > 5% and < 50% of infiltrating tumour cells presenting with a *HER2* copy number ≥ 6 (Pekar *et al.*, 2019; Vance *et al.*, 2009). Regional heterogeneity comprises an amplified tumour component admixed with a negative and/or equivocal tumour component based on immunohistochemistry and ISH studies (Bartlett *et al.*, 2011; Cottu *et al.*, 2008; Seol *et al.*, 2012). The observed heterogeneity suggests that in some tumours, not all cancer cells are depending on the *HER2* oncogene. Other genomic aberrations might act as potent alternative drivers of cancer cell proliferation and invasion in HER2‐negative subclones, such as the previously identified *BRF2* and *DSN1* gene amplification and the *HER2* p.I767M somatic mutation (Ng *et al.*, 2015).

In the current study, we aimed to further explore the landscape of somatic mutations and copy number variations (CNVs) in HER2‐heterogeneous breast cancers. We performed an in‐depth analysis of ten breast cancers containing at least two distinct components with different HER2 expression and copy number profiles, designated regional HER2 heterogeneity. We investigated whether these immunohistochemically distinct components were clonally related and whether the HER2‐negative components were associated with specific molecular aberrations that might act as alternative drivers of carcinogenesis.

## 2. Materials and methods

### 2.1. Patient samples

This retrospective study collected formalin‐fixed, paraffin‐embedded (FFPE) tissue samples from 10 breast cancer patients who were treated between 2010 and 2018 at the Erasmus Medical Center Cancer Institute (Rotterdam, the Netherlands). Coded leftover patient material was used in accordance with the Code of Conduct of the Federation of Medical Scientific Societies in the Netherlands (FEDERA, 2011), as previously described (Agahozo *et al.*, 2019). The study methodologies conformed to the standards set by the Declaration of Helsinki. The study methodologies were approved by the local ethics committee. Both core biopsies and resection specimens were eligible. Any histological type of breast cancer was included, provided that the tumour presented with regional heterogeneous *HER2* amplification and corresponding heterogeneous HER2 protein overexpression, as previously described (Seol *et al.*, 2012). Heterogeneous HER2 status was defined as the presence of at least one HER2‐positive *in situ* and/or invasive component and at least one HER2‐negative *in situ* and/or invasive component, as demonstrated by immunohistochemical and *in situ* hybridization (ISH) analysis. These different components had to be in close proximity of one another: all components were present in a single tissue block (with the exception of axillary lymph node metastases, if present). One representative tissue block was selected for all subsequent analyses.

### 2.2. Immunohistochemistry

Four‐µm‐thick FFPE tissue sections were mounted on Superfrost plus slides (Menzel‐Gläser, Braunschweig, Germany). Immunohistochemical stainings for oestrogen receptor (ER), progesterone receptor (PR), the myoepithelial cell marker p63, E‐cadherin, HER2, FGFR1 and EGFR were performed using an automatic immunostainer (Benchmark XT; Ventana Medical Systems, Tucson, AZ, USA), according to the manufacturer's instructions (Table S1). HER2 expression was assessed according to the ASCO/CAP guidelines (Wolff *et al.*, 2018). ER expression and PR expression were scored as percentages, regardless of the intensity. Hormone receptor status was determined according to the ASCO/CAP guidelines (Hammond *et al.*, 2010). Surrogate molecular intrinsic subtyping was based on the combined ER/PR/HER2 status.

### 2.3. HER2 *in situ* hybridization analysis

Automated HER2 ISH analysis was performed on all cases using the BenchMark ULTRA (Ventana Medical Systems). Four‐µm‐thick FFPE tissue sections were deparaffinized and incubated with cell conditioning 2 (CC2) buffer at 86 °C for 28 min. Tissue sections were treated with ISH‐Protease‐3 at 36 °C for 12 min, followed by HER2 probe denaturation at 96 °C for 8 min and hybridization at 80 °C for 6 min. UltraView SISH was used for detection, and haematoxylin II was used as counterstain. Tumour components were considered *HER2*‐amplified when a mean *HER2* copy number of ≥ 6 per cell was observed, in accordance with the ASCO/CAP guidelines (Wolff *et al.*, 2018).

### 2.4. DNA extraction

All tissue sections were first reviewed by two breast pathologists (MRVB and CHMVD) who selected tumour areas with an estimated minimum tumour cell percentage of 30%. Ten consecutive FFPE 5‐µm‐thick tissue sections were deparaffinized and haematoxylin‐stained prior to microdissection. Selected tumour areas and normal tissue areas were microdissected manually into 5% Chelex 100 Resin (Bio‐Rad, Hercules, CA, USA) Cell lysis solution (Promega, Madison, WI, USA), using a sterile scalpel. DNA was extracted by proteinase K (Roche, Mannheim, Germany) digestion by overnight incubation at 56 °C. Proteinase K was inactivated at 95 °C for 10 min. Finally, the samples were centrifuged for 5 min at 20 000 ***g*** to remove remaining cell debris and Chelex resins. The DNA was collected into new tubes and stored at −80 °C until further use. DNA concentrations were measured by a Qubit 2.0 fluorometer (Thermo Fisher Scientific, Waltham, MA, USA).

### 2.5. Targeted next‐generation sequencing

For targeted next‐generation sequencing (NGS), a custom‐made amplicon panel was applied. This panel comprised 2778 amplicons covering 53 genes (Table S2), including single nucleotide polymorphisms (SNPs) and hotspot mutation regions. Gene selection for this panel was based on two large tumour profiling studies (ICGC/TCGA and METABRIC), as well as frequently found driver mutations in breast cancer (Koboldt *et al.*, 2012; Nik‐Zainal *et al.*, 2016). The Ion AmpliSeq Designer tool was used to design amplicons for the multiplex PCR assay, thereby aiming for 150‐bp amplicons and allowing efficient amplification of fragmented DNA isolated from FFPE tissue. Full sequence coverage of large exons required amplification and sequencing of overlapping amplicons. Therefore, the multiplexed PCR was split into two reactions, using 10 ng of DNA for each reaction. The Ion AmpliSeq Library Kit Plus (Thermo Fisher Scientific) protocol was used to process the samples analysed by the Ion AmpliSeq custom 53‐gene panel, according to the manufacturer's instructions. Each sample was barcoded using IonXpress barcode adapters, allowing multiplexed sequencing. A total of 18 PCR cycles were performed. Ten samples were multiplexed on an Ion 540 Chip and sequenced on the Ion S5XL Semiconductor Sequencer (Thermo Fisher Scientific, Waltham, MA, USA).

### 2.6. Mutation analysis

The variant caller v5.6.0.4 (Thermo Fisher Scientific) was used for variant calling. Filtering was performed by the ‘somatic low stringency’ default of the Torrent Variant Caller. Variants were annotated in a local Galaxy pipeline (http://www.galaxyproject.org) using annovar (Wang *et al.*, 2010). Exonic and splice site variations were selected for analysis. Synonymous point mutations, as well as variants identified as common polymorphisms in the 1000 Genomes database (with a frequency of > 1%), were removed from the dataset. Variants were kept in the dataset if they had a minimum read depth of 100 reads and if they were present within a tumour component with a frequency higher than 10%. Variants were excluded if a strand artefact was suspected (forward/reverse or reverse/forward ratio of < 1/10). For each case, a patient‐matched normal tissue sample was analysed to verify whether the identified variants were somatic or germline. Pathogenic and likely pathogenic variants were considered germline if their variant allele frequency ranged within 45–55% in the normal tissue sample.

Four prediction algorithms, mutationtaster (http://www.mutationtaster.org/), provean (http://provean.jcvi.org/index.php), umd‐predictor (http://umd-predictor.eu/) and sift (https://sift.bii.a-star.edu.sg/), were used to predict the effects of coding nonsynonymous variants. The Catalogue of Somatic Mutations in Cancer (COSMIC; https://cancer.sanger.ac.uk/cosmic) was interrogated to assess for previous reports on the selected variants. Variants were selected when at least three of the four prediction algorithms indicated that the variant was pathogenic or probably pathogenic. If this criterion was not met, the variant was retained only if the COSMIC database indicated it was a known pathogenic or likely pathogenic variant. All variants were reported at the cDNA level (c. annotation) and the protein level (p. annotation) according to the Human Genome Variation Society (HGVS) nomenclature (Richards *et al.*, 2015).

### 2.7. Copy number variation analysis

The presence of high‐level gene copy number gains was investigated by using the relative coverage, as previously described (Eijkelenboom *et al.*, 2019). Sample normalization was performed to correct for differences in the number of total reads. The normal tissue samples of all patients constituted the reference series. The normalized coverage of the reference series was calculated by dividing the number of reads for each amplicon by the total number of reads for each normal tissue sample. The arithmetic mean was calculated for each amplicon, based on all samples in the reference series. The coverage of each amplicon from the tumour tissue samples was normalized by dividing the number of reads by the total number of reads per tumour tissue sample. The relative coverage for each amplicon of the tumour tissue samples was calculated by dividing the normalized coverage of the sample by the mean normalized coverage of the reference series (Eijkelenboom *et al.*, 2019). Copy number gains were suspected when at least five amplicons clustered together, provided that the log2 scale of the relative coverage amounted > 1.5. Copy number losses were not investigated as the presence of background (due to the use of FFPE tissue samples) hampered reliable interpretation of the presence of copy number losses. Visualization of CNVs was achieved by the construction of scatter plots in MS Office Excel (Windows, Washington, WA, USA).

## 3. Results

### 3.1. Patient population

**Fig. 1:**
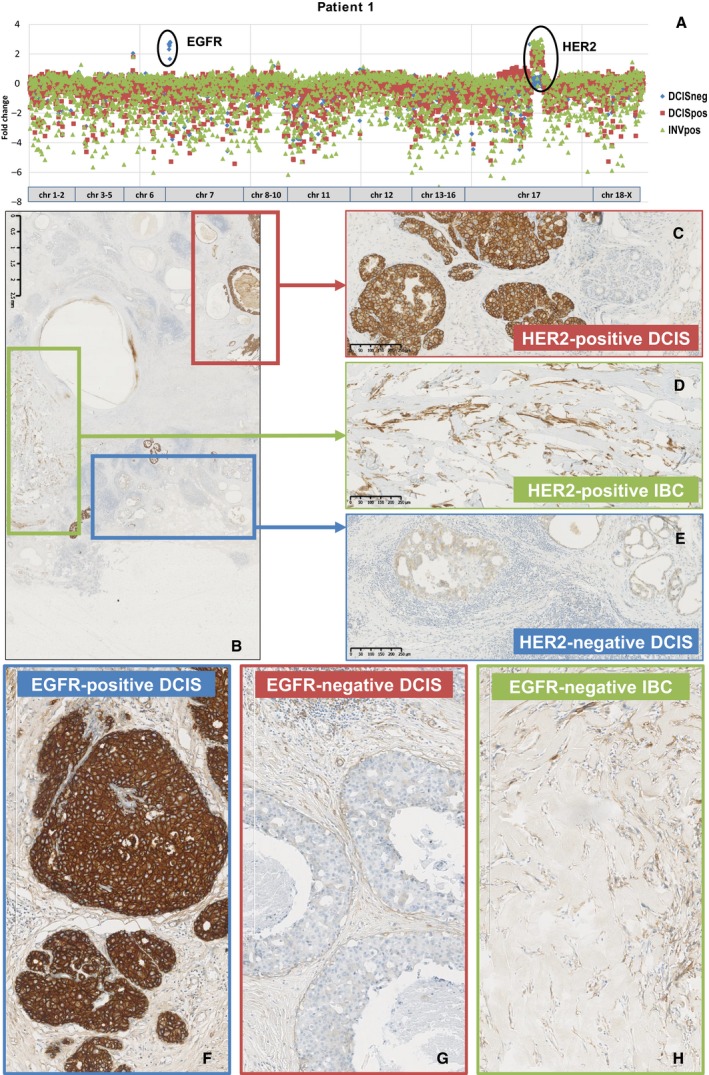
Heterogeneous CNVs and heterogeneous HER2 and EGFR expression in the tumour of patient #1. The scatter plot illustrates the presence of a HER2 copy number gain in one DCIS component and in the MC, as well as an EGFR copy number gain in the HER2‐negative DCIS component (A). Immunohistochemistry for HER2, with an overview of breast cancer #1 (B; original magnification 12.5× – scale bar size = 2.5 mm), and detailed microphotographs of the HER2‐positive DCIS (C), the HER2‐positive MC (D) and the HER2‐negative DCIS (E; original magnification 100× – scale bar size = 250 µm). Immunohistochemistry for EGFR, which was positive in the HER2‐negative DCIS component (F) and negative in the HER2‐positive DCIS (G) and in the HER2‐positive MC (H; original magnification 100× – scale bar size 300 µm).

**Fig. 2:**
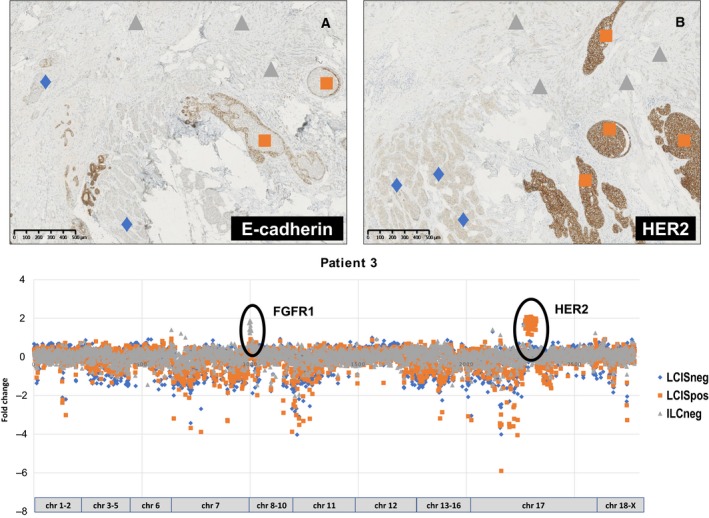
Heterogeneous HER2 overexpression and CNVs in the breast cancer of patient #3. Immunohistochemistry for E‐cadherin (A) illustrates the absence of expression in all tumour components (original magnification 50× – scale bar size = 500 µm). Immunohistochemistry for HER2 (B) demonstrates a positive 3+ score in the *HER2*‐amplified LCIS component (orange squares) and an equivocal 2+ score in the *HER2* nonamplified LCIS component (blue diamond) and the ILC of classic type (grey triangle; original magnification 50x – scale bar size = 500 µm). The scatter plot confirms the *HER2* copy number gain in the HER2‐positive LCIS (indicated by orange squares) and its absence in the HER2‐negative components (C). Additionally, the presence of an *FGFR1* copy number gain in the ILC is noted (indicated by grey triangles).

Ten patients with a breast cancer with spatially heterogeneous *HER2* amplification were included in this study. This series included eight patients with invasive carcinoma of no special type (NST) and associated ductal carcinoma *in situ* (DCIS), one patient with invasive lobular carcinoma (ILC) and associated lobular carcinoma *in situ* (LCIS), and one patient with metaplastic carcinoma (MC; spindle cell type) and associated DCIS. Figure 1 illustrates the presence of a HER2‐positive and a HER2‐negative DCIS component, associated with a HER2‐positive invasive MC (patient #1). Figure 2 demonstrates the presence of a HER2‐positive and HER2‐negative LCIS component associated with a HER2‐negative invasive component (patient #3). In some patients, heterogeneous *HER2* amplification was associated with heterogeneous hormone receptor status as well (Fig. S1; Table 1). All patients underwent nodal staging. Six patients had no sentinel lymph node metastases. Patient #10 had seven axillary macrometastases, with sufficient tissue available for targeted NGS. Patients #4, #9 and #5 had a sentinel lymph node with isolated tumour cells, a single micrometastasis and a single macrometastasis, respectively. These metastases were not analysed due to insufficient amounts of available tumour tissue.

**Table 1:** Detailed patient and tumour characteristics. Hormone receptor status and HER2 receptor status are indicated for each *in situ* and invasive tumour component. ER+, oestrogen receptor‐positive; ER−, oestrogen receptor‐negative; ID, patient pseudonym; NAC, neoadjuvant chemotherapy; NST, invasive carcinoma of no special type; PR+, progesterone receptor‐positive; PR−, progesterone receptor‐negative.

ID	Patient age at diagnosis (years)	Nottingham grade	Invasive tumour size (mm)^a^	Tumour and node stage	DCIS grade	Analysed specimen	NAC	*In situ* component *Hormone receptor status*		Invasive component *Hormone receptor status*	
								HER2‐negative	HER2‐positive	HER2‐negative	HER2‐positive
1	50	3	7	pT1bN0	3	Resection	No	DCIS *ER− PR−*	DCIS *ER− PR−*	–	MC *ER− PR−*
2	35	3	16	ypT1c(2) N0	3	Resection	Yes^b^	–	DCIS *ER− PR−*	NST *ER+ PR+*	NST *ER− PR−*
3	51	2	15	pT1c N0	–	Resection	No	LCIS *ER+ PR+*	LCIS *ER+ PR+*	ILC *ER+ PR+*	–
4	51	2	11	pT1c N0(i+)	3	Resection	No	–	DCIS *ER+ PR+*	NST *ER+ PR+*	NST *ER+ PR+*
5	55	3	24	pT2 N1a	3	Resection	No	DCIS *ER+ PR+*	–	NST *ER+ PR+*	NST *ER+ PR+*
6	50	2	12	ypT1c N0	3	Biopsy	Yes	–	DCIS^c^ *ER+ PR−*	NST *ER+ PR+*	NST *ER− PR−*
7	51	1	21	pT2 N0	2	Resection	No	–	DCIS *ER+ PR−*	NST *ER+ PR+*	–
8	56	3	18	pT1c N0	3	Resection	No	DCIS *ER+ PR+*	DCIS *ER+ PR−*	NST *ER+ PR*	NST *ER+ PR−*
9	55	2	18	pT1c N1(mi)	3	Resection	No	DCIS^c^ *ER+ PR+*	DCIS *ER+ PR*	NST *ER+ PR+*	–
10	42	3	19	pT1c N2b	3	Resection	No	–	DCIS *ER− PR−*	NST *ER− PR−* Axillary metastasis *ER− PR−*	–

a As measured in the resection specimen.

b Miller–Payne response grade 3

c Single duct, which disappeared during tissue sectioning; not included in this study because of insufficient material for targeted sequencing.

### 3.2. Coverage and mutation analysis

Sufficient DNA for sequencing was extracted from all but two microdissected tissue samples (Table 1). The mean percentage of amplicons with at least 100 and 500 reads was 94.4% and 78.5%, respectively, with an average base coverage depth of 2216 (Table S3). No pathogenic or likely pathogenic somatic variants were detected in *ARID1B, BRCA2, CCND3, CHECK2, ERBB2, ERBB3, MAP2K4* *, MLL, NCOR1, NOTCH1, PBRM1* and *PDGFRA*. Overall, germline pathogenic variants were not observed.

**Fig. 3:**
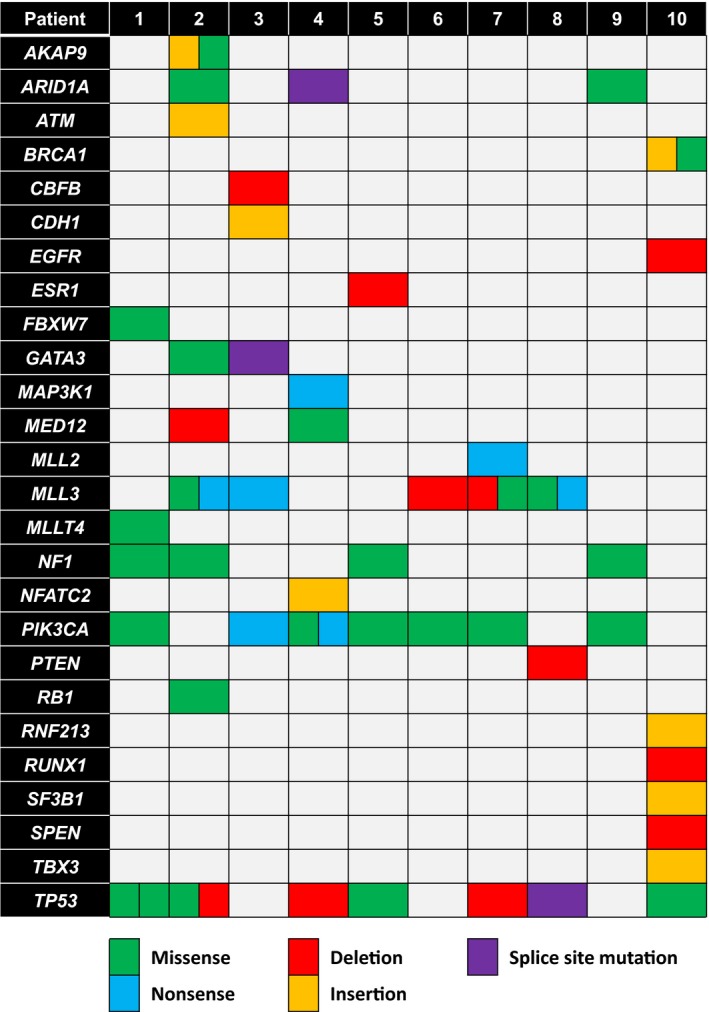
Overview of detected pathogenic and likely pathogenic variants in 26 genes per patient. Green and blue squares indicate missense and nonsense mutations, respectively. Red and orange squares indicate deletions and insertions, respectively. Splice site mutations are indicated by purple squares.

We identified 63 pathogenic or probably pathogenic variants in 26 different genes (Table S4), based on four prediction tools and the COSMIC database. These variants included 12 deletions, 9 insertions, 32 missense variants, 7 nonsense variants (with introduction of a stop codon) and 3 splice site alterations. These somatic aberrations were commonly found in *ARID1A, MLL3, NF1, PIK3CA* and *TP53* (Fig. 3). The tumour suppressor gene *TP53* was mutated in at least one component in 7 out of 10 breast cancers (70%). The *TP53* aberrations included five missense variants, two deletions and one splice site change. The presence of a *TP53* mutation was homogeneously present in all components of the breast cancers of patients #4, #5 and #8. Patients #1, #2, #7 and #10 each presented with a tumour with heterogeneous presence of a *TP53* mutation (Table 2). Patient #1 presented a p.R248W *TP53* mutation in the HER2‐negative DCIS component and the HER2‐positive invasive component, which was absent in the HER2‐positive DCIS component. However, the latter presented with a p.Y234H *TP53* mutation. Patient #2 presented with a p.S241fs deletion in both the HER2‐positive DCIS and the HER2‐positive invasive component, whereas the HER2‐negative DCIS component harboured a p.R273C missense variant. Patient #7 showed a p.R209fs *TP53* deletion in the HER2‐positive DCIS component, which was not detected in the associated HER2‐negative invasive component. Patient #10 showed a p.D259V missense variant in the HER2‐negative invasive component, which was not detected in the HER2‐positive DCIS, nor in the HER2‐negative axillary metastasis.

**Table 2:** Detailed information on the presence of somatic mutations in the more frequently mutated genes per patient and per tumour component. Chr: chromosome; FS: frameshift; ID: identity; Pt: patient pseudonym

Pt	Gene	Chr	Exon	Genbank transcript ID	Mutation	Protein change	COSMIC ID	Mutation consequence	FS	Presence in *in situ* component		Presence in invasive component	
										HER2−	HER2+	HER2−	HER2+
1	*MLL3*	7	7	NM_170606	c.980G>T	p.C327F	COSM340281	Missense	No	Absent	Absent	–	Present
1	*PIK3CA*	3	10	NM_006218	c.1624G>A	p.E542K	COSM760	Missense	No	Present	Present	–	Present
1	*TP53*	17	7	NM_000546	c.700T>C	p.Y234H	COSM2744649	Missense	No	Absent	Present	–	Absent
1	*TP53*	17	7	NM_000546	c.742C>T	p.R248W	COSM10656	Missense	No	Present	Absent	–	Present
2	*ARID1A*	1	3	NM_006015	c.1778C>T	p.S593F	–	Missense	No	–	Absent^a^	Absent^a^	Present
2	*MLL3*	7	7	NM_170606	c.851G>A	p.R284Q	COSM1179107	Missense	No	–	Present	Absent	Present
2	*MLL3*	7	36	NM_170606	c.6439C>T	p.Q2147X	–	Nonsense	Yes	–	Absent	Present	Absent
2	*RB1*	13	8	NM_000321	c.748C>T	p.P250S	COSM5730807	Missense	No	–	Absent^a^	Present	Absent
2	*RB1*	13	8	NM_000321	c.784C>T	p.R262W	COSM5047280	Missense	No	–	Absent^a^	Present	Absent
2	*TP53*	17	7	NM_000546	c.723delC	p.S241fs	COSM45831	Deletion	Yes	–	Present	Absent	Present
2	*TP53*	17	8	NM_000546	c.817C>T	p.R273C	COSM10659	Missense	No	–	Absent	Present	Absent
3	*MLL3*	7	14	NM_170606	c.2447dupA	p.Y816_I817delinsX	–	Nonsense	Yes	Absent	Absent	Present	–
3	*PIK3CA*	3	21	NM_006218	c.3145G>C	p.G1049R	COSM12597	Missense	No	Absent	Absent	Present	–
4	*ARID1A*	1	12	NM_006015	c.3425_3430AGGGC	p.?	–	Splice site change	?	–	Present	Present	Present
4	*PIK3CA*	3	21	NM_006218	c.3140A>G	p.H1047R	COSM775	Missense	No	–	Present	Present	Present
4	*PIK3CA*	3	21	NM_006218	c.3170G>A	p.W1057X	–	Nonsense	Yes	–	Absent	Present	Absent
4	*TP53*	17	6	NM_000546	c.578delA	p.H193fs	COSM45856	Deletion	Yes	–	Present	Present	Present
5	*PIK3CA*	3	21	NM_006218	c.3140A>G	p.H1047R	COSM775	Missense	No	Present	–	Present	Present
5	*TP53*	17	8	NM_000546	c.824G>A	p.C275Y	COSM10893	Missense	No	Present	–	Present	Present
6	*MLL3*	7	43	NM_170606	c.10224_10225del	p.Q3408fs	–	Deletion	Yes	–	–	Absent	Present
6	*PIK3CA*	3	10	NM_006218	c.1636C>G	p.Q546E	COSM6147	Missense	No	–	–	Present	Absent
7	*MLL3*	7	20	NM_170606	c.3258delC	p.S1086fs	–	Deletion	Yes	–	Absent	Present	–
7	*MLL3*	7	14	NM_170606	c.2468T>C	p.I823T	COSM6506545	Missense	No	–	Present	Present	–
7	*PIK3CA*	3	10	NM_006218	c.1633G>A	p.E545K	COSM760	Missense	No	–	Present	Present	–
7	*TP53*	17	6	NM_000546	c.626_627del	p.R209fs	COSM45817	Deletion	Yes	–	Present	Absent	–
8	*MLL3*	7	7	NM_170606	c.851G>A	p.R284Q	COSM1179107	Missense	No	Absent	Present	Present	Absent
8	*MLL3*	7	14	NM_170606	c.2447dupA	p.Y816_I817delinsX	–	Nonsense	Yes	Present	Absent	Absent	Absent
8	*TP53*	17	(intron)	NM_000546	c.782+1G>T	p.?	COSM473431	Splice site change		Present	Present	Present	Present
9	*ARID1A*	1	3	NM_006015	c.1778C>T	p.S593F	–	Missense	No	–	Absent	Present	–
9	*PIK3CA*	3	21	NM_006218	c.3140A>G	p.H1047R	COSM775	Missense	No	–	Present	Present	–
10	*TP53*	17	7	NM_000546	c.776A>T	p.D259V	COSM43724	Missense	No	–	Absent	Present^b^	–

a Low coverage of the corresponding amplicon (i.e. < 100 reads).

b The mutation was not detected in the HER2‐negative axillary metastasis.

Seven out of ten (70%) breast cancers harboured a *PIK3CA* mutation in at least one tumour component. Patients #1, #4, #5, #7 and #9 presented with a breast cancer with homogeneous presence of a *PIK3CA* mutation in each individual tumour component, whereas *PIK3CA* mutations were heterogeneously distributed in the tumours of patients #3 and #6 (Table 2). Patient #3 showed a p.G1049R missense mutation in the HER2‐negative invasive component, which was not detected in the DCIS components, irrespective of their HER2 status. Patient #6 showed a p.Q546E *PIK3CA* mutation which was present in the HER2‐negative invasive component and absent in the admixed HER2‐positive invasive component. Patient #4 had a p.H1047R *PIK3CA* mutation in each tumour component, but the HER2‐negative invasive component harboured an additional p.W1057X mutation, which was not detected in the other tumour components. Somatic *ARID1A*, *MLL3* and *NF1* mutations were found in at least one component in three, six and four breast tumours, respectively, and the presence of these mutations was unrelated to the HER2 status (Fig. S2, Table 2). For instance, patient #1 showed a p.C327F *MLL3* mutation in the HER2‐positive carcinoma component, which was absent in both DCIS components. Patient #3 had a nonsense mutation in the HER2‐negative invasive component, which was lacking in both DCIS components. Patient #8 had a missense *MLL3* mutation in the HER2‐positive DCIS and HER2‐negative invasive component, whereas the HER2‐negative DCIS component presented with a different nonsense *MLL3* mutation.

Less common somatic variants were observed in *AKAP9, ATM, BRCA1, CBFB, CDH1, EGFR, ESR1, FBXW7, GATA3, MAP3K1* *, MED12, MLL2, MLLT4, NFATC2, PTEN, RB1, RNF213, RUNX1, SF3B1, SPEN* and *TBX3* (Table S5). Somatic mutations in these genes were often heterogeneously present throughout the different tumour components, and their presence seemed unrelated to the *HER2* amplification status, except for *GATA3* mutations. Somatic *GATA3* mutations occurred in HER2‐positive tumour components. Patient #2 had a *GATA3* mutation in the HER2‐positive *in situ* and invasive components, which was absent in the HER2‐negative invasive carcinoma component. Patient #3 had a *GATA3* splice site mutation in the HER2‐positive LCIS component, which was not observed in the HER2‐negative *in situ* and invasive components.

### 3.3. Copy number variation analysis

The presence of high‐level CNVs was investigated, and confirmed the presence of *HER2* amplification in all HER2‐positive carcinoma samples, which served as an internal quality control (Figs 1 and 2; Figs S1 and S2). Additionally, we observed an *EGFR* copy number gain in the HER2‐negative DCIS component of patient #1 (Fig. 1). Patients #3 and #5 had an *FGFR1* copy number gain in at least one tumour component. In patient #3, the HER2‐negative ILC harboured this *FGFR1* amplification (Fig. 2), which was absent in the HER2‐negative and HER2‐positive LCIS components. In patient #5, all tumour components displayed the *FGFR1* copy number gain. Patients #9 and #10 both had a HER2‐negative tumour component with a gain of 8q24, which comprised a copy number gain of both *MYC* and the adjacent long noncoding RNA (lncRNA) plasmacytoma variant translocation 1 (*PVT1*). Patient #9 also had a *CCND1* copy number gain in the HER2‐negative tumour component. The potential presence of copy number losses was difficult to interpret with certainty, as some amplicons showed a consistently lower coverage throughout this series. The use of FFPE samples caused a relatively high background, which further hampered the assessment of any potentially relevant deletions.

### 3.4. Complementary immunohistochemical analysis

Immunohistochemistry for EGFR was performed on all tumour tissue samples. In patient #1, the identified EGFR amplification in the HER2‐negative DCIS component was associated with EGFR protein overexpression (Fig. 1F–H). No EGFR protein overexpression was noted in the other tumours (data not shown). Immunohistochemistry for FGFR1 was performed on tumour tissue samples of patients #3 and #5, which revealed no apparent positivity in either of the tumour components (data not shown).

## 4. Discussion

Carcinogenesis is an evolutionary process governed by the principles of Darwinian dynamics (Gillies *et al.*, 2012). Tumours are clonal proliferations, originating from a single cell that acquired genomic instability through an accumulation of somatic mutations. Early genomic anomalies, including crucial oncogenic drivers, will therefore be present in all tumour cells and constitute clonal molecular aberrations. Acquisition of additional oncogenic drivers and passenger mutations will result in subpopulations of cancer cells with different genotypes and phenotypes, and these subclonal aberrations contribute to intratumour heterogeneity (McDonald *et al.*, 2019). This heterogeneity is caused by somatic mutations and CNVs, as well as differences in epigenetics (Assenov *et al.*, 2018; Easwaran *et al.*, 2014). Somatic evolution is driven by a combination of genetic instability and a selective tumour microenvironment, including acidosis, hypoxia and cytotoxic stress imposed by chemotherapy, hormonal therapy and/or targeted therapies (Gillies *et al.*, 2012). HER2‐targeted therapies impose an evolutionary selection pressure on HER2‐positive cancer cells. Those cancer cell populations that are not exclusively dependent on the overexpression of the *HER2* oncogene will be able to constitute an anti‐HER2 therapy‐resistant subclone, regardless of their HER2 status. These subclones harbour alternative and/or collaborative drivers of carcinogenesis, which circumvent the blockade of the HER2‐driven pathways. The high prevalence of both intrinsic and acquired resistance to single‐agent treatment regimens already caused a shift towards dual HER2‐targeted therapy, such as pertuzumab or T‐DM1 (Konecny, 2013; Pernas *et al.*, 2018).

Interestingly, 5–41% of HER2‐positive breast cancers present with regional heterogeneous *HER2* amplification (Cottu *et al.*, 2008; Ng *et al.*, 2015), although this percentage depends on the applied definition. In this study, we subjected ten breast cancers with spatially heterogeneous *HER2* amplification and corresponding HER2 overexpression to targeted NGS. We investigated the potential heterogeneity in the somatic mutational landscape of each individual tumour component. Some mutations were, if present, homogeneously found in each component. For instance, four of seven tumours with a *PIK3CA* mutation presented this mutation in each component. Somatic *TP53* mutations seemed more often heterogeneously distributed, and their presence seemed generally unrelated to the *HER2* amplification status.

Two breast cancers in this series harboured a gain of the 8q24 region, comprising both *MYC* and the adjacent lncRNA *PVT1*, which stabilizes the MYC protein and enhances its activity (Tseng and Bagchi, 2015). Co‐amplified *MYC* and *PVT1* genes have been identified as candidate oncogenes in ER‐positive, HER2‐positive breast cancers (Sircoulomb *et al.*, 2010). A recent meta‐analysis concluded that increased PVT1 expression was associated with lower overall survival in a wide variety of solid tumours, including breast cancer (Zou *et al.*, 2019). High PVT1 expression was associated with clinicopathological markers of poor prognosis, such as larger tumour size, higher TNM stage and the presence of both lymph node and distant metastases (Zou *et al.*, 2019). *In vitro* studies demonstrated that PVT1 expression drives cancer cell proliferation through promotion of the KLF5/BAP1/beta‐catenin signalling pathway (Tang *et al.*, 2018).

One patient had a *CCND1* copy number gain in a HER2‐negative invasive tumour component. *CCND1* amplification is associated with a particular gene expression profile and decreased survival in ER‐positive, HER2‐negative node‐negative breast cancer patients (Lundberg *et al.*, 2019), indicating that *CCND1* amplification might act as an alternative driver of carcinogenesis. Similar observations have been reported for *FGFR1* amplification within breast cancer and other types of carcinoma (Helsten *et al.*, 2016).

By using targeted NGS with a 53‐gene panel, we identified a plethora of somatic mutations and CNVs within the HER2‐negative components in this series of ten HER2 heterogeneous breast cancers. The genetic heterogeneity within both the HER2‐negative and HER2‐positive components of a single tumour suggests that a wide range of different somatic mutations and/or CNVs may act as potential alternative drivers. These genetic aberrations might counterbalance the absence of *HER2* amplification in the HER2‐negative components. Of note, this targeted NGS‐driven study focussed only on a subset of 53 breast cancer‐related genes in a limited series of ten breast cancer patients. Since we did not apply whole‐genome sequencing on a large patient series, it is impossible to exclude the existence of a more commonly present alternative driver in HER2‐negative tumour components. Due to the use of FFPE material, we were confronted with high levels of background in some tumour tissue samples, which precluded an in‐depth analysis of potentially important copy number losses. Nevertheless, our findings are in accordance with the observations of the TCGA network, who described a high frequency of *TP53* (55%) and *PIK3CA* (31%) mutations, and an low frequency of mutations in *RUNX1* (1%), *PTEN* (0%), *NCOR* (0%) and *CDH1* (3%) in 75 clinically HER2‐positive breast cancers (Koboldt *et al.*, 2012). The TCGA network identified a high frequency of *TP53* mutations in ER‐negative, HER2‐positive breast cancers, whereas ER‐positive, HER2‐positive breast cancers displayed more often a *GATA3* mutation (Koboldt *et al.*, 2012). This hormone receptor‐dependent duality was not observed in our series, which might be due to its small size.

The limited gene panel precludes strong statements regarding the clonal relationship of all components within a single tumour. However, the integration of histopathological and immunohistochemical features, together with the uniform presence of some well‐defined pathogenic mutations (such as *TP53* or *PIK3CA* mutations), suggests a common progenitor for most heterogeneous lesions in this series. Based on the frequent homogeneous presence of the observed variants, it was estimated that the tumours of patients #1, #3, #4, #5, #7 and #9 were likely to have a common progenitor. The tumours of patients #2, #6 and #10 were considered to be less likely related to one another (i.e., a collision tumour of two independent neoplastic lesions), or to have a common progenitor with very early divergence of the subclones. Despite its limited size, this series of ten breast cancers demonstrates that regional heterogeneity in HER2 status is associated with further heterogeneity at the molecular level, and sometimes also at the protein level, since some tumour components presented with different hormone receptor status and/or EGFR protein expression status. Although regional HER2 heterogeneity is uncommon, this series illustrates that not all cells within one tumour depend exclusively on *HER2* amplification and overexpression.

Due to its relatively high prevalence in invasive breast cancer and its association with worse prognosis, HER2 overexpression is suspected to play a major role as a driver of mammary carcinogenesis. HER2‐positive invasive breast cancer more often presents with an associated *in situ* component, and if present, this DCIS component is substantially larger than in HER2‐negative tumours (Doebar *et al.*, 2016). The prevalence of HER2 overexpression amounts 35% in pure DCIS, which is paradoxically higher than its prevalence in invasive breast cancer (Siziopikou *et al.*, 2013). Overall, HER2 expression profiles are highly concordant between admixed *in situ* and invasive breast cancer, but overexpression/amplification is less common in the DCIS component of admixed lesions than in pure DCIS (Burkhardt *et al.*, 2010; Lambein *et al.*, 2017; Latta *et al.*, 2002; Park *et al.*, 2006). One in three women with a HER2‐positive pure DCIS lesion develops a subsequent HER2‐negative invasive breast cancer (Visser *et al.*, 2019), although the clonal relationship between primary and recurrent lesions was not investigated in that study. Taken together, these observations indicate that HER2 overexpression is more likely to play a role as an instigator of tumour cell proliferation, rather than being a crucial driver of cancer cell invasion (Sanati, 2019). The series of pathogenic and likely pathogenic somatic variants that we describe here yields a wide range of potential alternative drivers of cancer cell proliferation and invasion. Moreover, some genetic anomalies (such as *PIK3CA* and *GATA3* mutations, or *FGFR1* copy number gain) might drive resistance to treatment (Pernas *et al.*, 2018; Turner *et al.*, 2010).

## 5. Conclusion

The HER2‐negative components of HER2 heterogeneous breast cancers display a variety of somatic mutations and CNVs within 53 breast cancer‐related genes. Although these somatic mutations and CNVs were often present in the HER2‐positive component as well, they might act as potential alternative drivers to counterbalance the absence of *HER2* amplification. Since these potential alternative drivers may have the capacity to circumvent HER2 pathway blockade, their widespread presence throughout these HER2 heterogeneous cancers might explain the high level of innate and acquired resistance to HER2‐targeted therapies in breast cancer. Our findings indirectly imply that a targeted monotherapy is unlikely to have high efficacy in the long term, since it causes cytotoxic distress and selection of those resistant clones that already harbour alternative drivers of carcinogenesis. Future translational breast cancer research should focus on how to handle this molecular heterogeneity in the clinical setting.

## Conflict of interest

MRVB is supported by the Mathilde Horlait‐Dapsens Foundation (Brussels, Belgium) and the non‐for‐profit organization Foundation Against Cancer (Grant 2019‐089, Brussels, Belgium). The other authors report that they have no conflicts of interest to disclose.

## Author contributions

MRVB and MCA performed the mutation analysis and copy number variation analysis, and designed the figures and tables of the manuscript. MRVB and CHMD performed the histopathological review and interpreted immunohistochemical and SISH analyses. RM, PNA and WNMD were responsible for performing tissue microdissection, DNA isolation, library preparation and targeted next‐generation sequencing. HFBMS was responsible for HER2 ISH analysis. EHL, LLV and JW designed the breast‐specific gene panel used for targeted next‐generation sequencing. CHMD was responsible for the study design and patient selection. MRVB wrote the first draft of the manuscript. All contributors reviewed and edited the manuscript, and approved its final version.

## Supporting information


**Fig. S1.** Copy number variations, HER2 status and hormone receptor status in the breast cancer of patient #6. The scatter plot confirms the presence of a *HER2* copy number gain in the HER2‐positive invasive carcinoma component (A; indicated by red squares). Immunohistochemistry for HER2 (B), oestrogen receptor (C) and progesterone receptor (D) illustrate opposite protein expression profiles in both invasive carcinoma components (original magnification 12,5x – scale bar size = 2,5 mm).

**Fig. S2.** Copy number variations in the breast cancers of patients #5, #9 and #10. The scatter plot of patient #5 (A) confirms a *HER2* copy number gain in the HER2‐positive invasive carcinoma component (indicated by green triangles), and demonstrates an *FGFR1* copy number gain (cytogenetic location: 8p11.23) in each tumour component. The tumour of patient #9 harbours a neighbouring copy number gain located at 8q24 in all carcinoma components, which comprises both the *MYC* and *PVT1* genes, as well as a *CCND1* copy number gain in the HER2‐negative invasive carcinoma component (B). A similar 8q24 copy number gain was noted in the HER2‐negative invasive carcinoma component (indicated by red triangles) of patient #10 (C). In patient #9, this co‐amplification was present in both the HER2‐positive DCIS and the HER2‐negative invasive carcinoma components, indicating that this genetic aberration can occur as an early event in carcinogenesis. However, this co‐amplification was not present in the HER2‐positive DCIS component and the HER2‐negative axillary metastasis.

**Table S1.** Materials and methods for immunohistochemistry.

**Table S2.** List of genes included in the panel used for targeted next‐generation sequencing.

**Table S3.** Detailed information on base coverage and number of reads for targeted next‐generation sequencing of normal and tumour tissue samples of ten breast cancers with regional HER2 heterogeneity.

**Table S4.** Mutation analysis.

**Table S5.** Detailed information on the presence of somatic mutations in the less frequently mutated genes per patient and per tumour component.

## Data Availability

All data generated and/or analysed during the current study are available from the corresponding author on reasonable request.
