# Supplementary figures and images for: Somatic mutations and copy number variations in breast cancers with heterogeneous HER2 amplification
Source: Mol Oncol. 2020 Mar 5;14(4):671–85. doi: 10.1002/1878-0261.12650 (PMC7138394; doi:10.1002/1878-0261.12650)

# PATIENT 6

A

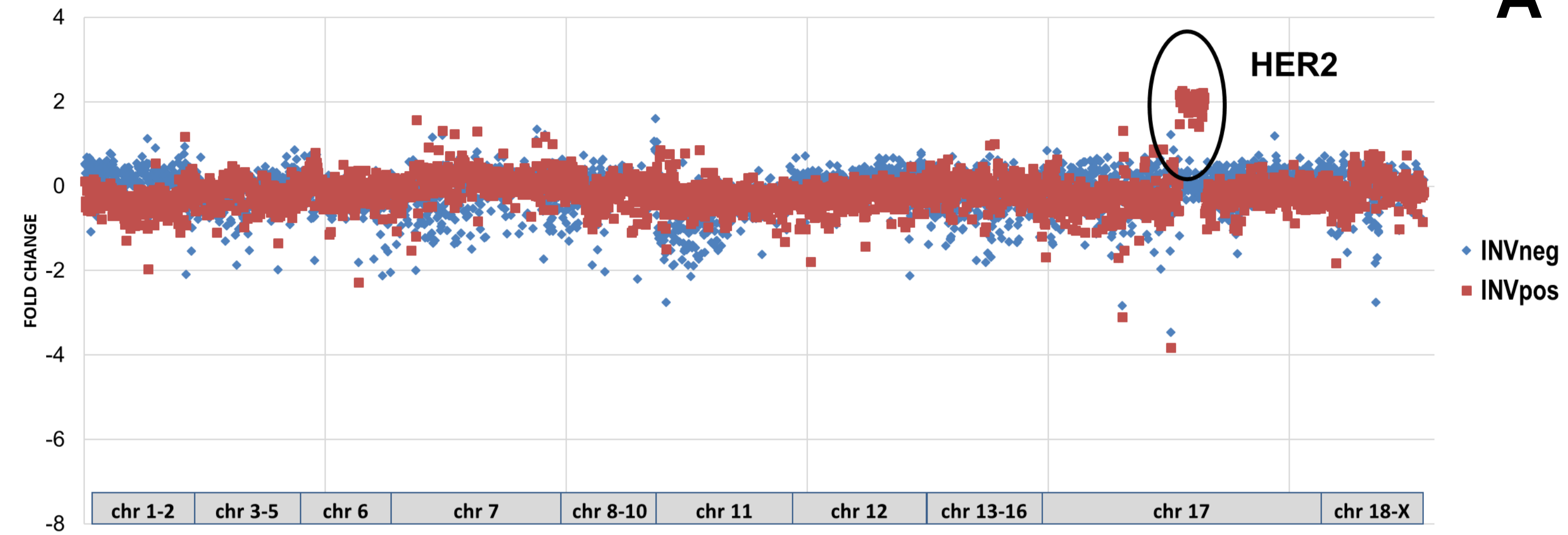

B

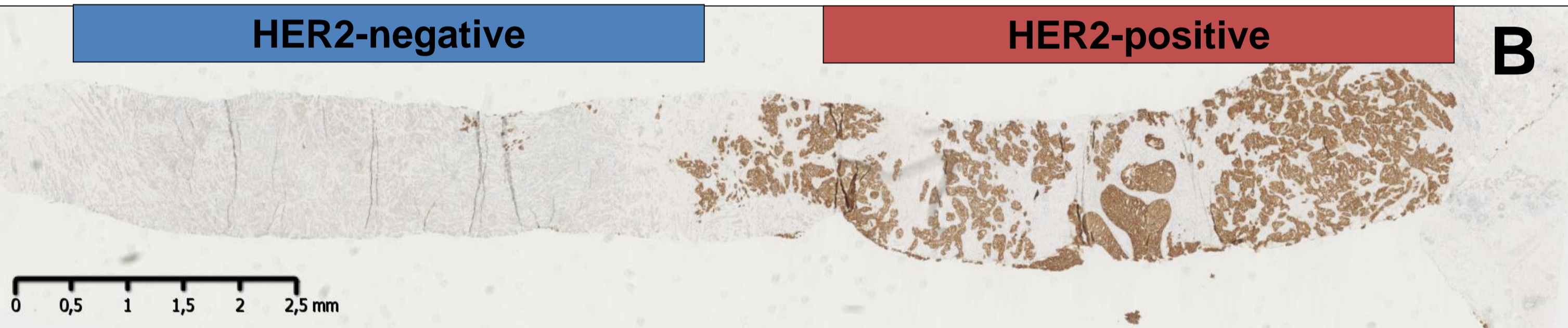

C

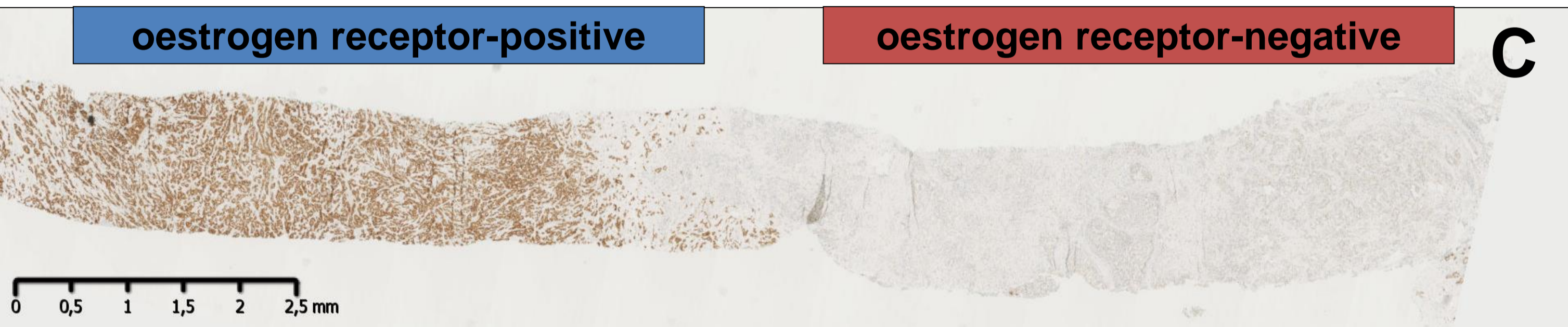

D

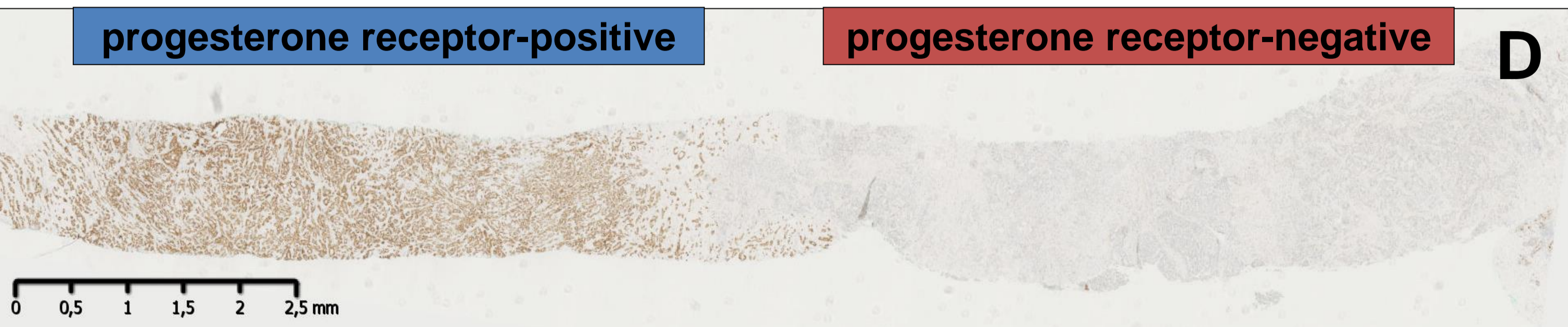

Supplement: Supplementary file 1 — Fig. S1. Copy number variations, HER2 status and hormone receptor status in the breast cancer of patient #6. The scatter plot confirms the presence of a HER2 copy number gain in the HER2‐positive invasive carcinoma component (A; indicated by red squares). Immunohistochemistry for HER2 (B), oestrogen receptor (C) and progesterone receptor (D) illustrate opposite protein expression profiles in both invasive carcinoma components (original magnification 12,5x – scale bar size = 2,5 mm). [file MOL2-14-671-s001.pdf]

PATIENT 5

A

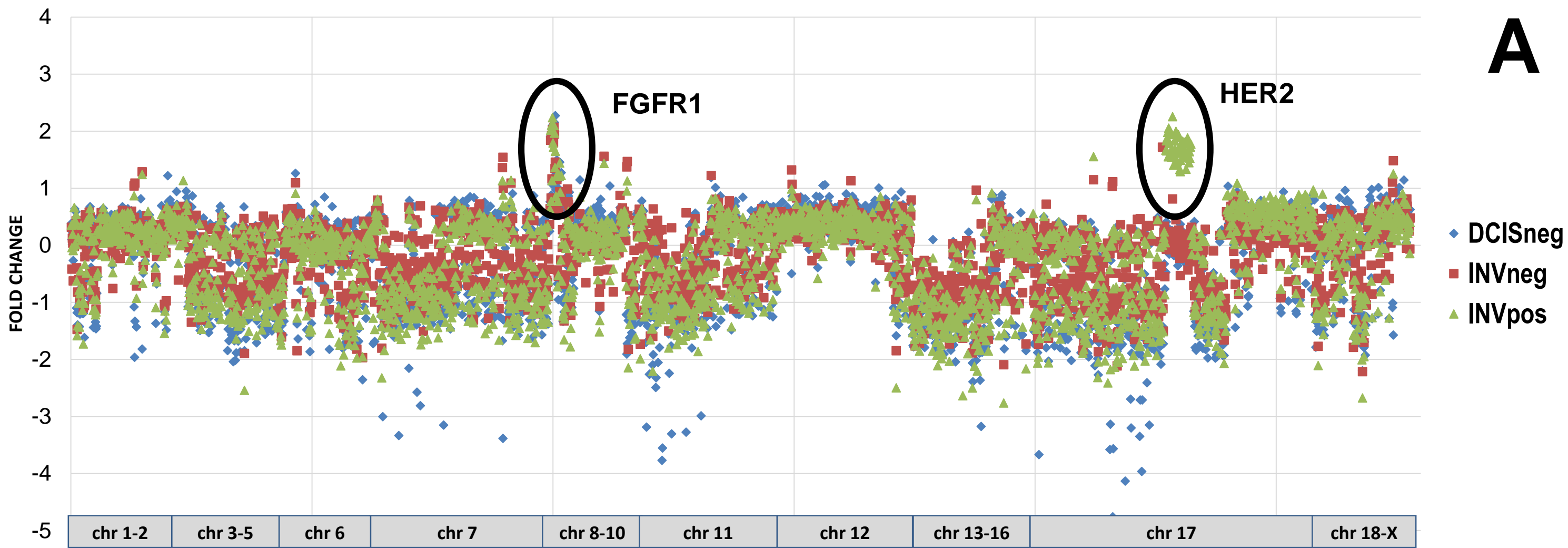

PATIENT 9

B

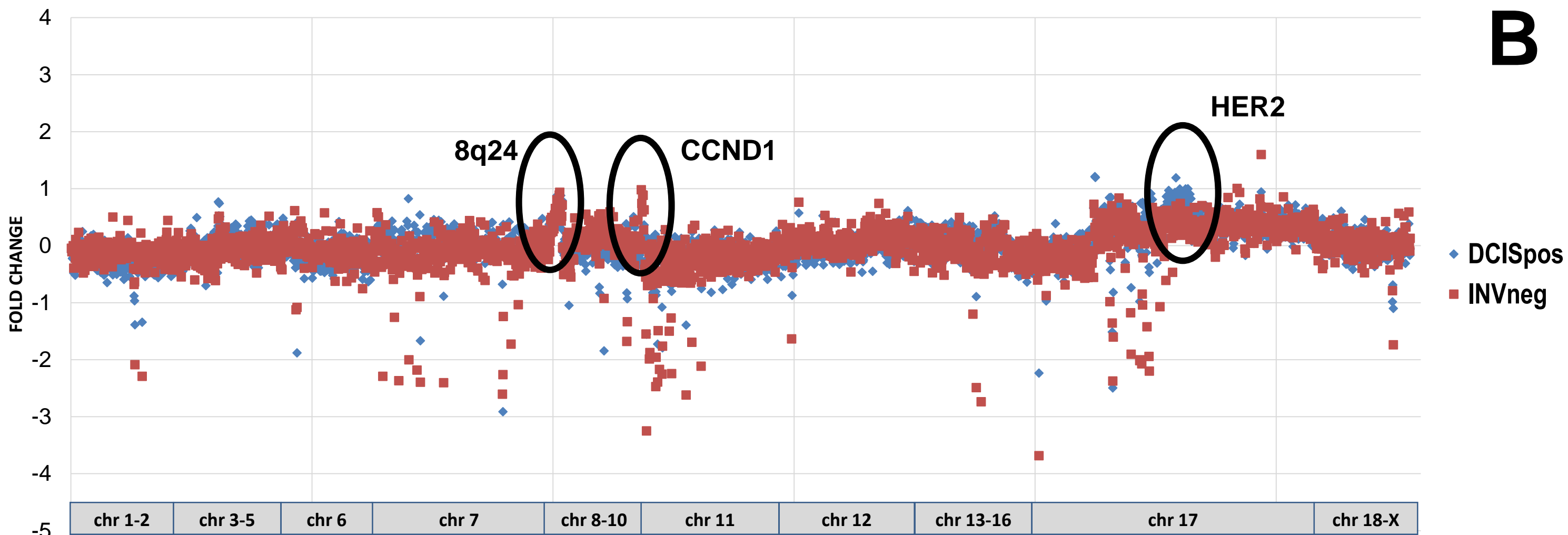

PATIENT 10

C

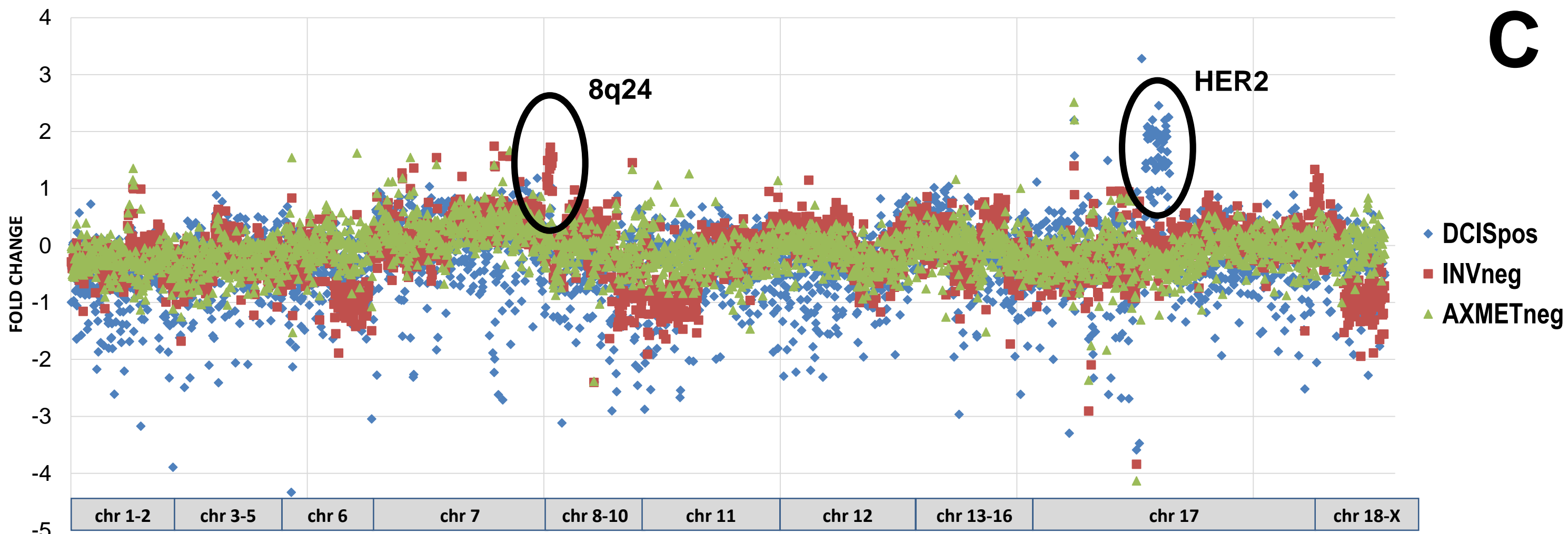

Supplement: Supplementary file 2 — Fig. S2. Copy number variations in the breast cancers of patients #5, #9 and #10. The scatter plot of patient #5 (A) confirms a HER2 copy number gain in the HER2‐positive invasive carcinoma component (indicated by green triangles), and demonstrates an FGFR1 copy number gain (cytogenetic location: 8p11.23) in each tumour component. The tumour of patient #9 harbours a neighbouring copy number gain located at 8q24 in all carcinoma components, which comprises both the MYC and PVT1 genes, as well as a CCND1 copy number gain in the HER2‐negative invasive carcinoma component (B). A similar 8q24 copy number gain was noted in the HER2‐negative invasive carcinoma component (indicated by red triangles) of patient #10 (C). In patient #9, this co‐amplification was present in both the HER2‐positive DCIS and the HER2‐negative invasive carcinoma components, indicating that this genetic aberration can occur as an early event in carcinogenesis. However, this co‐amplification was not present in the HER2‐positive DCIS component and the HER2‐negative axillary metastasis. [file MOL2-14-671-s002.pdf]
