# Supplementary material for: Somatic mutations and copy number variations in breast cancers with heterogeneous HER2 amplification
Source: Mol Oncol. 2020 Mar 5;14(4):671–85. doi: 10.1002/1878-0261.12650 (PMC7138394; doi:10.1002/1878-0261.12650)
Supplement: Supplementary file 3 — Table S1. Materials and methods for immunohistochemistry. [file MOL2-14-671-s003.docx]

| **Supplementary Table 1. Materials and methods for immunohistochemistry.** | | | | | | | |
| --- | --- | --- | --- | --- | --- | --- | --- |
| **Antigen** | **Antibody clone** | **Host species primary antibody** | **Manufacturer** | **Dilution** | **HIER procedure** | **Incubation time primary antibody** | **Secondary antibody** |
| Estrogen receptor | SP1 | Rabbit | Ventana/Roche | RTU | CC1 – 64 minutes | 32 minutes | Ultraview |
| Progesterone receptor | 1E2 | Rabbit | Ventana/Roche | RTU | CC1 – 36 minutes | 12 minutes | Ultraview |
| HER2 | 4B5 | Rabbit | Ventana/Roche | RTU | CC1 – 36 minutes | 32 minutes | Ultraview |
| p63 | 4A4 | Mouse | Ventana/Roche | RTU | CC1 – 64 minutes | 32 minutes | Optiview |
| E-cadherin | 36 | Mouse | Ventana/Roche | RTU | CC1 – 32 minutes | 32 minutes | Optiview |
| EGFR | L8A4 | Mouse | Absolute Antibody | 1:200 | CC1 – 64 minutes | 32 minutes | Ultraview |
| FGFR1 | M2F12 | Mouse | Abcam | 1:100 | CC1 – 64 minutes | 32 minutes | Optiview |
| CC1: Cell Conditioning 1; EGFR: epidermal growth factor receptor; FGFR1: fibroblast growth factor receptor 1; HIER: heat-induced epitope retrieval; RTU: ready-to-use | | | | | | | |
