# Supplementary material for: Somatic mutations and copy number variations in breast cancers with heterogeneous HER2 amplification
Source: Mol Oncol. 2020 Mar 5;14(4):671–85. doi: 10.1002/1878-0261.12650 (PMC7138394; doi:10.1002/1878-0261.12650)
Supplement: Supplementary file 5 — Table S3. Detailed information on base coverage and number of reads for targeted next‐generation sequencing of normal and tumour tissue samples of ten breast cancers with regional HER2 heterogeneity. [file MOL2-14-671-s005.docx]

| **Supplementary Table 3. Detailed information on base coverage and number of reads for targeted next-generation sequencing of normal and tumour tissue samples of ten breast cancers with regional *HER2* heterogeneity.** | | | | | | | |
| --- | --- | --- | --- | --- | --- | --- | --- |
| **Patient** | **Tissue component*** | **Uniformity of base coverage**  **(%)** | **Percent reads on target**  **(%)** | **Amplicons with at least 100 reads**  **(%)** | **Amplicons with at least 500 reads**  **(%)** | **Number of mapped reads** | **Average base coverage depth** |
| 1 | Normal | 88,28 | 94,85 | 95,82 | 86,11 | 6078194 | 2179 |
| 1 | DCISneg | 87,75 | 94,48 | 95,03 | 85,10 | 5824110 | 2072 |
| 1 | DCISpos | 78,31 | 94,34 | 90,78 | 74,15 | 5481058 | 1910 |
| 1 | INVpos | 64,74 | 94,36 | 80,27 | 58,35 | 5074948 | 1732 |
| 2 | Normal | 82,68 | 91,78 | 96,47 | 86,39 | 10025501 | 3242 |
| 2 | DCISpos | 69,62 | 93,70 | 84,05 | 60,48 | 4409961 | 1444 |
| 2 | INVneg | 59,18 | 91,52 | 67,64 | 49,93 | 4016225 | 1248 |
| 2 | INVpos | 71,78 | 94,75 | 86,00 | 63,32 | 4748795 | 1601 |
| 3 | Normal | 89,66 | 93,43 | 98,67 | 95,68 | 15458763 | 5433 |
| 3 | LCISneg | 87,26 | 93,63 | 97,44 | 87,08 | 7720501 | 2746 |
| 3 | LCISpos | 90,18 | 93,60 | 97,34 | 92,19 | 8504719 | 3040 |
| 3 | INVneg | 94,93 | 93,06 | 98,56 | 95,82 | 8084802 | 2827 |
| 4 | Normal | 94,52 | 92,68 | 97,26 | 88,80 | 4186999 | 1387 |
| 4 | DCISpos | 93,71 | 93,44 | 98,52 | 93,45 | 6970194 | 2373 |
| 4 | INVneg | 89,50 | 94,32 | 94,20 | 65,84 | 2596273 | 901 |
| 4 | INVpos | 90,55 | 94,02 | 93,92 | 52,77 | 2113011 | 731 |
| 5 | Normal | 90,16 | 92,47 | 94,74 | 59,47 | 2440388 | 830 |
| 5 | DCISneg | 78,93 | 94,73 | 89,99 | 57,34 | 2681654 | 971 |
| 5 | INVneg | 87,40 | 94,18 | 98,31 | 87,54 | 7446156 | 2648 |
| 5 | INVpos | 81,75 | 95,34 | 98,27 | 82,87 | 7845897 | 2872 |
| 6 | Normal | 88,48 | 92,22 | 97,05 | 90,42 | 8691226 | 2746 |
| 6 | INVneg | 90,53 | 93,92 | 83,59 | 18,72 | 949324 | 324 |
| 6 | INVpos | 93,30 | 93,18 | 97,25 | 86,14 | 4605543 | 1536 |
| 7 | Normal | 97,11 | 92,69 | 99,24 | 97,95 | 12247741 | 4127 |
| 7 | DCISpos | 88,84 | 91,45 | 96,15 | 85,96 | 8262244 | 2765 |
| 7 | INVneg | 88,31 | 90,67 | 95,63 | 84,72 | 7723536 | 2542 |
| 8 | Normal | 95,27 | 92,24 | 98,74 | 96,72 | 11672278 | 3896 |
| 8 | DCISneg | 95,61 | 93,28 | 98,92 | 96,18 | 8194519 | 2808 |
| 8 | DCISpos | 94,65 | 93,54 | 98,31 | 96,00 | 10322507 | 3585 |
| 8 | INVneg | 95,34 | 93,15 | 97,95 | 95,72 | 8479271 | 2926 |
| 8 | INVpos | 95,53 | 94,59 | 99,06 | 97,12 | 10235938 | 3629 |
| 9 | Normal | 95,09 | 92,55 | 98,49 | 95,32 | 8124384 | 2736 |
| 9 | DCISpos | 94,74 | 92,45 | 98,63 | 95,18 | 7408619 | 2510 |
| 9 | INVneg | 93,63 | 92,40 | 98,24 | 94,46 | 8349903 | 2827 |
| 10 | Normal | 95,08 | 91,88 | 97,88 | 77,83 | 3162727 | 1026 |
| 10 | DCISpos | 85,35 | 90,29 | 87,01 | 34,81 | 1631621 | 502 |
| 10 | INVneg | 91,64 | 91,05 | 95,50 | 57,70 | 2401653 | 775 |
| 10 | mINVneg | 93,71 | 90,07 | 97,16 | 57,52 | 2385315 | 746 |
| * DCIS: ductal carcinoma in situ; INV: invasive carcinoma; LCIS: lobular carcinoma in situ; mINV: metastasis of invasive carcinoma; neg: HER2-negative; pos: HER2-positive. | | | | | | | |
