# Supplementary material for: Somatic mutations and copy number variations in breast cancers with heterogeneous HER2 amplification
Source: Mol Oncol. 2020 Mar 5;14(4):671–85. doi: 10.1002/1878-0261.12650 (PMC7138394; doi:10.1002/1878-0261.12650)
Supplement: Supplementary file 7 — Table S5. Detailed information on the presence of somatic mutations in the less frequently mutated genes per patient and per tumour component. [file MOL2-14-671-s007.docx]

| **Supplementary Table 5.** **Detailed information on the presence of somatic mutations in the less frequently mutated genes per patient and per tumour component.** | | | | | | | | | | | | | |
| --- | --- | --- | --- | --- | --- | --- | --- | --- | --- | --- | --- | --- | --- |
| **Pt** | **Gene** | **Chr** | **Exon** | **Genbank Transcript ID** | **Mutation** | **Protein change** | **COSMIC ID** | **Mutation consequence** | **FS** | **Presence in in situ component** | | **Presence in invasive component** | |
|  |  |  |  |  |  |  |  |  |  | **HER2-** | **HER2+** | **HER2-** | **HER2+** |
| **1** | *FBXW7* | 4 | 4 | NM_033632 | c.629G>T | p.G210V | - | Missense | No | Absent | Present | - | Present |
| **1** | *MLLT4* | 6 | 25 | NM_001207008 | c.3338A>G | p.E1113G | - | Missense | No | Absent | Absent | - | Present |
| **1** | *NF1* | 17 | 15 | NM_000267 | c.1658A>G | p.H553R | COSM98377 | Missense | No | Present | Absent | - | Present |
| **2** | *AKAP9* | 7 | 41 | NM_005751 | c.10129_10130TG | R3377Lfs | - | Insertion | Yes | - | Absent | Present | Absent |
| **2** | *AKAP9* | 7 | 41 | NM_005751 | c.10118C>T | p.S3373F | - | Missense | No | - | Absent | Present | Absent |
| **2** | *ATM* | 11 | 11 | NM_000051 | c.1681_1682TA | Q561Lfs | - | Insertion | Yes | - | Absent ^$^ | Present | Absent |
| **2** | *GATA3* | 10 | 6 | NM_001002295 | c.1198C>T | p.H400Y | - | Missense | No | - | Present | Absent ^$^ | Present |
| **2** | *MED12* | X | 42 | NM_005120 | c.6237delC | I2079Nfs | - | Insertion | Yes | - | Absent | Present | Absent |
| **2** | *NF1* | 17 | 18 | NM_000267 | c.2224G>A | p.A742T | - | Missense | No | - | Absent | Present | Absent |
| **2** | *NF1* | 17 | 38 | NM_000267 | c.5627G>A | p.G1876D | - | Missense | No | - | Absent ^$^ | Present | Absent ^$^ |
| **2** | *NF1* | 17 | 38 | NM_000267 | c.5633G>A | p.C1878Y | - | Missense | No | - | Absent ^$^ | Present | Absent ^$^ |
| **2** | *NF1* | 17 | 44 | NM_000267 | c.6665C>T | p.S2222F | - | Missense | No | - | Present | Absent | Absent |
| **3** | *CBFB* | 16 | 3 | NM_022845 | c.170_180del | p.F57fs | - | Deletion | Yes | Present | Present | Present | - |
| **3** | *CDH1* | 16 | 3 | NM_004360 | c.204dupT | p.Y68fs | - | Insertion | Yes | Present | Present | Present | - |
| **3** | *GATA3* | 10 | (intron) | NM_001002295 | c.925-3delCA | p.? | COSM166053 | Splice site change | Yes | Absent | Present | Absent | - |
| **4** | *MAP3K1* | 5 | 14 | NM_005921 | c.3352G>T | p.E1118X | - | Nonsense | Yes | - | Present | Present | Present |
| **4** | *MED12* | X | 26 | NM_005120 | c.3592G>A | p.G1198R | - | Missense | No | - | Present | Absent | Absent ^$^ |
| **4** | *NFATC2* | 20 | 4 | NM_012340 | c.1503_1504CC | p.L503Pfs | - | Insertion | Yes | - | Present | Present | Absent |
| **5** | *ESR1* | 6 | 5 | NM_001122741 | c.975delG | p.P325fs |  | Deletion | Yes | Absent | - | Present | Present |
| **5** | *NF1* | 17 | 15 | NM_000267 | c.1658A>G | p.H553R | COSM98377 | Missense | No | Absent | - | Absent | Present |
| **7** | *MLL2* | 12 | 48 | NM_003482 | c.15061C>T | p.R5021X | COSM5704469 | Nonsense | Yes | - | Present | Present | - |
| **8** | *PTEN* | 10 | 8 | NM_000314 | c.950_953del | p.V317fs | COSM5347162 | Deletion | Yes | Present | Present | Absent | Absent |
| **8** | *PTEN* | 10 | 8 | NM_000314 | c.955_958del | p.T319fs | - | Deletion | Yes | Absent | Present | Present | Present |
| **9** | *NF1* | 17 | 32 | NM_000267 | c.4312G>A | p.E1438K | - | Missense | No | - | Absent | Present | - |
| **10** | *BRCA1* | 17 | 10 | NM_007300 | c.1071dupA | p.L358fs | - | Insertion | Yes | - | Present | Absent ° | - |
| **10** | *BRCA1* | 17 | 10 | NM_007300 | c.3113A>G | p.E1038G | COSM3755562 | Missense | No | - | Present | Present* | - |
| **10** | *EGFR* | 7 | 13 | NM_005228 | c.1539delG | p.E513fs | - | Deletion | Yes | - | Absent | Present ° | - |
| **10** | *RNF213* | 17 | 26 | NM_001256071 | c.5552dupC | p.T1851fs | - | Insertion | Yes | - | Present | Absent ° | - |
| **10** | *RUNX1* | 21 | 4 | NM_001754 | c.178_183GCCCG | p.P61fs | - | Deletion | Yes | - | Absent | Present ° | - |
| **10** | *SF3B1* | 2 | 20 | NM_012433 | c.2931_2932AGT | p.L978fs | - | Insertion | Yes | - | Present | Absent ° | - |
| **10** | *SPEN* | 1 | 11 | NM_015001 | c.5849delG | p.G1950fs | - | Deletion | Yes | - | Absent | Present ° | - |
| **10** | *TBX3* | 12 | 7 | NM_005996 | c.2168dupC | p.P723fs | - | Insertion | Yes | - | Present | Absent ° | - |
| Chr: chromosome; FS: frameshift; ID: identity; Pt: patient  * The mutation was detected in the HER2-negative axillary metastasis  ^$^ Low coverage of the corresponding amplicon (i.e. <100 reads)  ° The mutation was not detected in the HER2-negative axillary metastasis. | | | | | | | | | | | | | |
